# Prognostic impact of the Controlling Nutritional Status score following curative nephrectomy for patients with renal cell carcinoma: Erratum

**DOI:** 10.1097/MD.0000000000014200

**Published:** 2019-01-11

In the article, “Prognostic impact of the Controlling Nutritional Status score following curative nephrectomy for patients with renal cell carcinoma”,^[1]^ which appears in Volume 97, Issue 49 of *Medicine*, the contribution note in the footnote should appear as “YZ, LB, and WW contributed equally to this manuscript.”
